# Understanding migraine in Saudi society: An assessment of public knowledge and attitudes: A cross-sectional study

**DOI:** 10.1371/journal.pone.0304840

**Published:** 2024-06-21

**Authors:** Fahad S. Alshehri, Ahmed M. Ashour, Adnan S. Alharbi, Alqassem Y. Hakami, Nasser M. Alorfi

**Affiliations:** 1 Pharmacology and Toxicology Department, College of Pharmacy, Umm Al-Qura University, Makkah, Saudi Arabia; 2 Pharmacy Practice Department, College of Pharmacy, Umm Al-Qura University, Makkah, Saudi Arabia; 3 College of Medicine, King Saud bin Abdulaziz University for Health Sciences, Jeddah, Saudi Arabia; 4 King Abdullah International Medical Research Center, Jeddah, Saudi Arabia; University of Hafr Al-Batin, SAUDI ARABIA

## Abstract

**Objective:**

This study aims to assess the knowledge and perceptions of the public toward migraine in Saudi Arabia.

**Methods:**

This cross-sectional survey assessed the knowledge and perceptions of migraine among Saudi Arabian individuals. The study was conducted over three months in 2023 (1^st^ of June 2023 to 31^st^ of August 2023) using a prevalidated online questionnaire divided into four sections.

**Results:**

A total of 1,975 adults aged between 18 and 64 completed the web-based survey. Of these, over half were male (n = 1,268; 64.2%). The main causes of migraine identified by the participants were genetic disease (n = 540, 27.3%), followed by physical disease (n = 341, 17.3%), head trauma (n = 274, 13.9%), and psychiatric disease (n = 157, 7.9%). The main symptoms identified by the participants were photophobia (21%), followed by inability to control urine (14.1%), vomiting and nausea (13.8%), and vision loss (8.3%). The majority of the participants in this study had a good knowledge of migraines, while 49% had poor knowledge. The migraine knowledge score was significantly associated with the participants’ gender (p = 0.002), age (p = 0.0001), educational level (p = 0.001), employment status (p = 0.001), monthly income (p = 0.0001), region (p = 0.0001), and history of migraine (p = 0.0001).

**Conclusion:**

Although one-third of the participants exhibiting good knowledge, deficiencies existed in certain clinical aspects, emphasizing the need for targeted educational interventions to enhance public awareness and understanding of migraines.

## Introduction

A migraine is a chronic neurological disorder that affects millions around the world every day and it is experienced by many individuals at some point in their lives [1–4]. In 2020, migraines were the second leading cause of disability globally [2], and it is the third most prevalent condition among individuals [3]. Worldwide, 10% of individuals suffer from migraine, mostly between the ages of 20 and 50 years [5]. The prevalence of migraine varies between countries and populations [5–10]. For instance, in the United States, it was 22.7% in 2022 [5] and some Saudi studies have estimated a prevalence of 25–37.2% in Saudi Arabia [8–10]. In Taif city, reports have revealed the prevalence of migraine to be around 22.6% in females and 7.2% in males [6, 11]. On the other hand, a recent systematic review of Arab countries in 2022 revealed a prevalence of 2.6%–32% [9]. Furthermore, it is believed that women are more likely than men to suffer migraines [5, 6, 12]. In Italy, the estimated prevalence of migraine was between 8.2% and 13.7% [7].

There is a link between migraines and sensitivity to light, sound, and movement during periods of headache [10, 13]. The occurrence of recurrent headaches, coupled with any number of neurological symptoms, is called "recurrent headache syndrome" [13]. Although the exact cause of migraine has yet to be determined, previous research has linked migraine to genetic or environmental factors [14]. Furthermore, hormonal changes, childbirth, amenorrhea, anxiousness and stress, coffee beverages, alcohol, changes in sleep, physical factors, changes in the weather or environment, food products and their additives, and several medications, including contraceptives, have been discovered to have a triggering effect on migraine [14, 15]. In addition to its sporadic attacks and loss of productivity [16], migraine has other severe negative effects on an individual’s life, which include disturbed quality of all aspects of life [8, 9], marital and family relationships, parenting life [17], abnormal work-life balance [7] and difficulties with financial achievement, stability, and overall health [16]. Furthermore, the treatment costs associated with migraine are believed to be high [17, 18].

Given the above consequences associated with migraine, it is essential to manage or treat it in order to reduce the personal and financial burden on the patient and healthcare systems. The available treatment options use both pharmacological and non-pharmacological treatments [19]. For mild to moderate migraines, non-prescription pain relievers like acetaminophen and ibuprofen are commonly used, while prescription triptans such as sumatriptan provide relief for more severe cases [20–22]. Preventive medications, including beta-blockers and anticonvulsants, may be prescribed for frequent or severe migraines [23, 24], along with newer drugs targeting the Calcitonin Gene-Related Peptide (CGRP) pathway [25, 26]. Lifestyle modifications, such as identifying triggers and maintaining regular sleep patterns, play a crucial role in managing migraines [27, 28]. Dietary changes, relaxation techniques, biofeedback, and physical therapy are non-pharmacological approaches that individuals may explore [29–31]. Acupuncture has also shown promise in reducing migraine frequency and intensity [32].

Adequate knowledge and awareness about the disease are crucial for further control of the severity of the disease [33–37]. There are few studies assessing the general public’s knowledge and perception of migraines in the Kingdom of Saudi Arabia (KSA) [1, 36]. However, most previous studies have revealed a poor level of knowledge and awareness of migraine in Saudi Arabia [1, 36, 38] and other countries [39]. This study aims to evaluate the awareness and perceptions of migraine symptoms among the general population in Saudi Arabia. Understanding how different demographic groups recognize and respond to migraines is critical for developing targeted interventions that can improve early detection and management, ultimately enhancing quality of life and reducing the disease’s impact.

## Materials and methods

The aim of this study was to assess migraine awareness and knowledge among the general population. The study was carried out with the general public living in Saudi Arabia, between 1^st^ of June 2023 and 31^st^ of August 2023, after receiving IRB approval form Umm Al-Qura’s Biomedical Research Ethics Committee (HAPO-02-K-012-2023-05-1606). Participants aged 18 years or older, living in Saudi Arabia, were eligible for inclusion in the study. Verbal consent was obtained from individuals stating a willingness to participate, while anyone not fitting the inclusion criteria was excluded.

An online sample size calculator was used to calculate the required representative sample size to achieve the study objectives and ensure sufficient statistical power. The calculator determined a target of 1,083 participants, margin of error of ±5%, a confidence level of 99.9%, a response distribution of 50%, and a population size of 36,947,025 people. A suitable questionnaire was adopted using the previous literature [12–15]. The questionnaire comprised four sections in both Arabic and English. Section one gathered the participants’ demographic characteristics with a total of 10 items (for example age, gender, nationality, and qualifications). The second section of the questionnaire dealt with the clinical perspective and individuals’ perceptions of migraine (cause, complications, symptoms, source of information, effect of gender, and age group). These items were collected via multiple choice options. The third section of the study dealt with migraine knowledge, and it was composed of 11 items, assessed on a three-point scale of True/False/I don’t know. The last section of the questionnaire dealt with the perceived barriers to proper management among sufferers. This item was assessed on a multiple-choice scale.

After the questionnaire had been drafted, it was subjected to expert review to evaluate its readability, content, and length of time to complete. Then, considering all the suggestions and comments, the questionnaire was employed in a pilot study with the help of randomly elected individuals. Before the questionnaire was administered, a reliability test was conducted to determine whether it was reliable, and the results revealed that the questionnaire had an acceptable level of internal consistency, based on Cronbach’s Alpha (Cronbach’s Alpha = 0.70) for the migraine knowledge questions.

Individuals were contacted and asked if they were willing to participate in the study. For data collection, electronic questionnaires were prepared, using Google Forms as the main platform. The invitation link and the electronic questionnaire were sent to the participants. The data were collected using a convenience sampling method until the required sample number was obtained. The data collection was completed by a primary investigator and a researcher. The estimated migraine prevalence in the population, desired level of precision, and estimated response rate was considered when calculating the target sample size. To obtain a maximum number of responses, follow-up messages were sent to the participants, along with weekly reminders to complete the questionnaires and send them back. The data collection followed the snowballing technique, where one referral or participant brings multiple referrals and participants to the study.

### Data analysis

The data were analyzed with SPSS 26 (SPSS Inc., Chicago, IL). The demographic characteristics were calculated using descriptive statistics, such as frequencies (n) and percentages (%). Then, univariate analysis (chi-squared test/exact Fisher’s test) was used to determine how the variables differed. All statistical analyses were conducted at a significance level of 0.05.

## Results

A total of 1,975 adults aged between 18 and 64 completed the web-based survey. Of these, the majority were male (n = 1,268; 64.2%) and young, aged 25–33 years (n = 737; 37.3%), while 30% (n = 593) were aged 18–24 years. The majority were Saudi nationals (n = 1,973; 99.9%). More than one-third of the adults reported a good monthly income (between 10,000 and 15,000) or an average monthly income (between 5,000 and 9,000) Saudi Riyals (SAR). More than half 54.4% (n = 1074) of the adults had been educated at university and 27.7% were students. Table 1 presents the demographic characteristics of the participants.

**Table 1:** Demographics and other characteristics of the study subjects (n = 1,975).

Characteristics	Frequency (n)	Percentage (%)
**Gender**		
Male	1268	64.2
Female	707	35.8
**Age (years)**		
18–24	593	30.0
25–33	737	37.3
34–51	524	26.5
52–64	77	3.9
>64	44	2.2
**Nationality**		
Saudi	1973	99.9
Non-Saudi	2	0.1
**Educational level**		
Secondary	478	24.2
University	1074	54.4
Postgraduate studies	219	11.1
Intermediate	204	10.3
**Employment status**		
Housewife	315	15.9
Businessman	463	23.4
Student	547	27.7
Unemployed	215	10.9
Retired	97	4.9
Employed	338	17.1
**Monthly income in SAR**		
Good (between 10,000 and 15,000)	729	36.9
Low (<5,000)	323	16.4
Average (5,000–9,999)	717	36.3
Excellent (>15,000)	206	10.4
**Region**		
Southern	575	29.1
Eastern	505	25.6
Northern	397	20.1
Western	346	17.5
Central	152	7.7
**History of migraine?**		
Yes	1350	68.4
No	625	31.6

**Fig 1:**
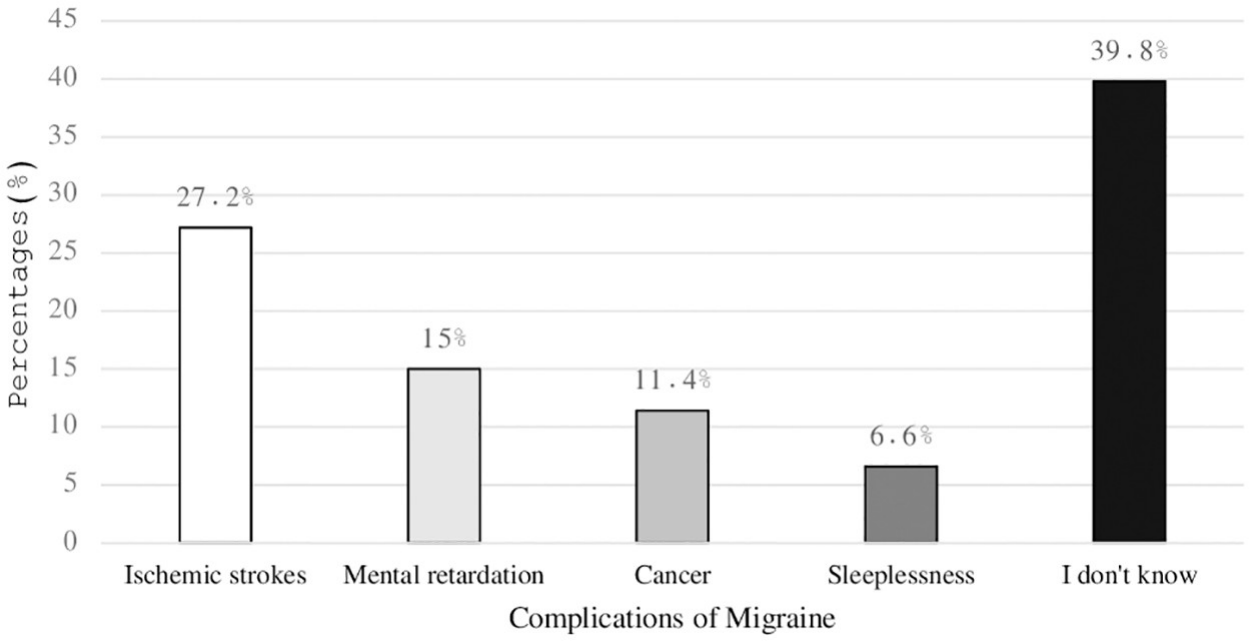
Complications of migraine.

The current findings revealed that more than two-thirds of the participants had a history of migraine. The main causes of migraine reported by the participants were genetic disease (27.3%), followed by physical disease (17.3%), head trauma (13.9%), and psychiatric disease (7.9%) (Table 2). Furthermore, over 30% of the participants were unaware of the complications (39.8%) and symptoms (35%) of migraine (Fig 1).

**Table 2:** Participants’ perceptions about the clinical aspects of migraine.

Variables	Frequency (n)	Percentage (%)
**Causes of migraine**		
Physical diseases	341	17.3
Genetic diseases	540	27.3
Psychiatric diseases	157	7.9
Head trauma	274	13.9
I don’t know	663	33.6
**Complications of migraine**		
Ischemic strokes	537	27.2
Mental retardation	296	15.0
Cancer	225	11.4
Sleeplessness	131	6.6
I don’t know	786	39.8
**Treatment for migraine**		
Medications	207	10.5
Herbal medicine	339	17.2
Surgery	319	16.2
Electrical shocks	139	7.0
Vitamins	211	10.7
I don’t know	669	33.9
There is no treatment	91	4.6
**Most common group affected by migraine**		
Children	152	7.7
Young	482	24.4
Elderly	1341	67.9
**Most common gender group affected by migraine**		
Females	218	11.0
Males	80	4.1
Both	970	49.1
I don’t know	707	35.8

**Fig 2:**
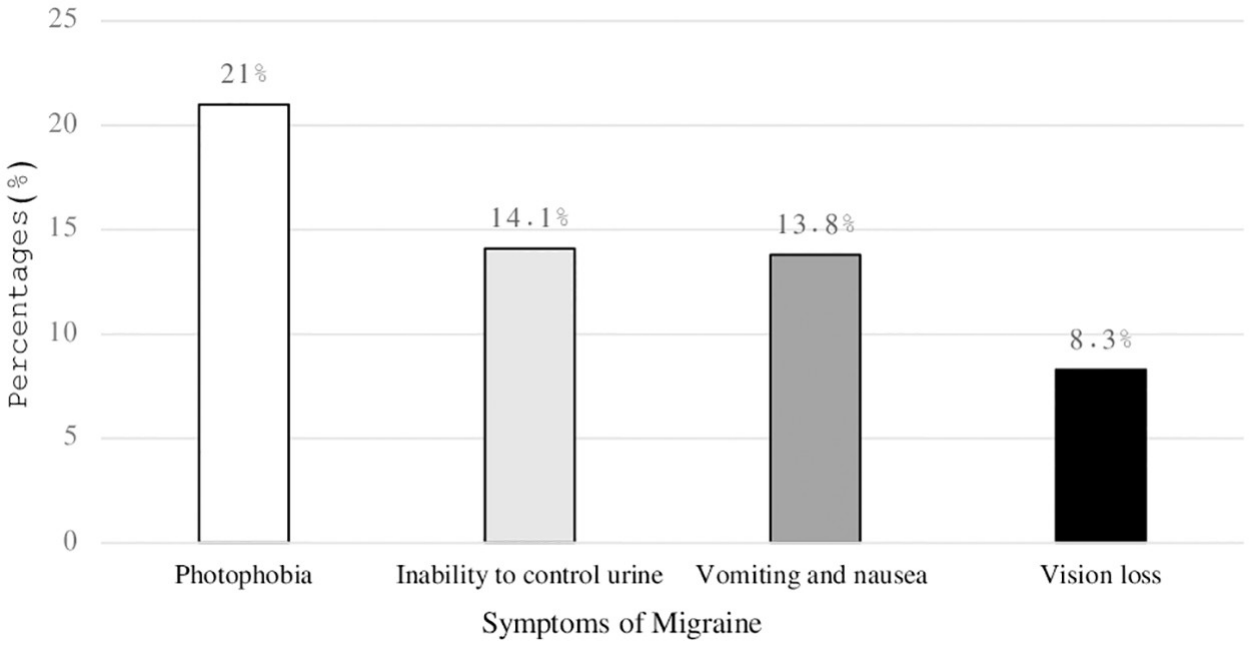
Symptoms of migraine.

However, photophobia was reported by 21% (n = 415), followed by an inability to control urine 14.1% (n = 279), vomiting and nausea 13.8% (n = 272), and vision loss 8.3% (n = 164) (Fig 2).

**Fig 3:**
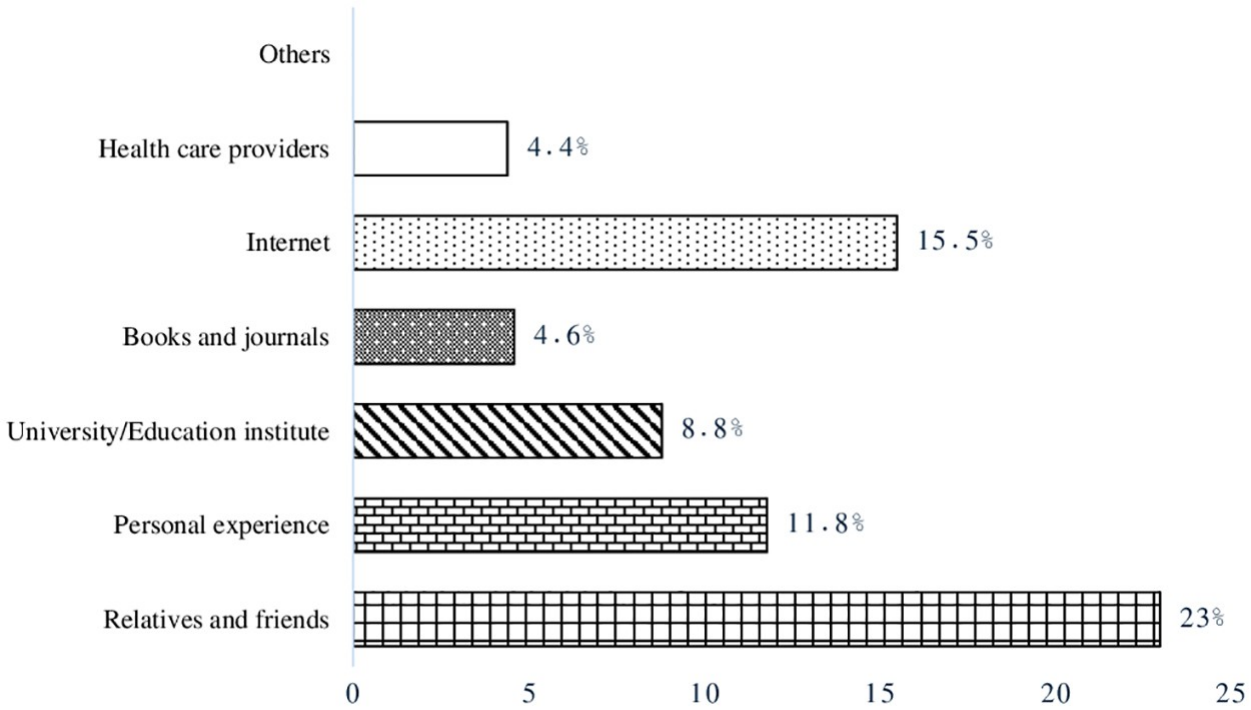
Sources of knowledge of migraine.

The most commonly stated sources of knowledge for migraine were relatives and friends (23%; n = 455) followed by the internet (15.5%; n = 306). Other sources included personal experiences (11.8%; n = 233), university/education institute (8.8%; n = 173), books and journals (4.6%; n = 91), and healthcare providers (4.4%; n = 87) (Fig 3).

In this study, over 30% of the participants claimed that they did not know how migraines were treated. On the other hand, 17.2% used herbal medicines, followed by vitamins (10.7%) and electrical shock (7%; n = 139). Most of them perceived the elderly (67.9%) as the most common age group affected by migraine and they believed that both genders (49.1%) were equally affected by migraines. The detailed information related to the participants’ perceptions is given in Table 2.

With regard to the participants’ knowledge about migraine, almost half of the participants (50.8%) believed that migraines usually affected one out of three females. Only 26.6% of the participants in this study agreed that headaches associated with migraines are usually bilateral and pulsating. Around 45% of the respondents knew that migraine relapses could last from four to 72 hours. Most of them were unaware of the fact that migraines have emotional, cognitive, and behavioral features. Seventy-three percent of the respondents did not know that stress and hormonal imbalances could cause relapses, and the majority did not know that loud noise and bright light could cause migraine relapses, while less than half of the participants (42.4%) were aware that extreme low and high temperatures did not trigger migraine attacks. Most of the participants (66.8%) were unaware that migraines could be treated with acute and preventive medications, and non-pharmacological approaches as well. Further information regarding their knowledge of migraines is provided in Table 3.

**Table 3:** Participants’ responses about their knowledge of migraine.

Variables	Yes	No
	n (%)	n (%)
Migraine usually affects 1 out of 3 individuals of female gender	1004 (50.8)	971 (49.2)
Clinically, headache associated with migraine is usually bilateral and pulsating	525 (26.6)	1450 (73.4)
Relapses of migraine may last between 4 and 72 h	887 (44.9)	1088 (55.1)
Migraine has emotional, cognitive, and behavioral features	539 (27.3)	1436 (72.7)
In the KSA, the majority of affected cases receive appropriate preventive treatment	884 (44.8)	1091 (55.2)
Stress and hormonal imbalance are risk factors for relapses	532 (26.9)	1443 (73.1)
Noise and bright light can trigger migraine relapse	519 (26.3)	1456 (73.7)
Extreme low or high temperatures are not triggers for migraine relapse	838 (42.4)	1137 (57.6)
Migraine can be treated with acute and preventive medications as well as a variety of non-pharmacological techniques	655 (33.2)	1320 (66.8)

**Fig 4:**
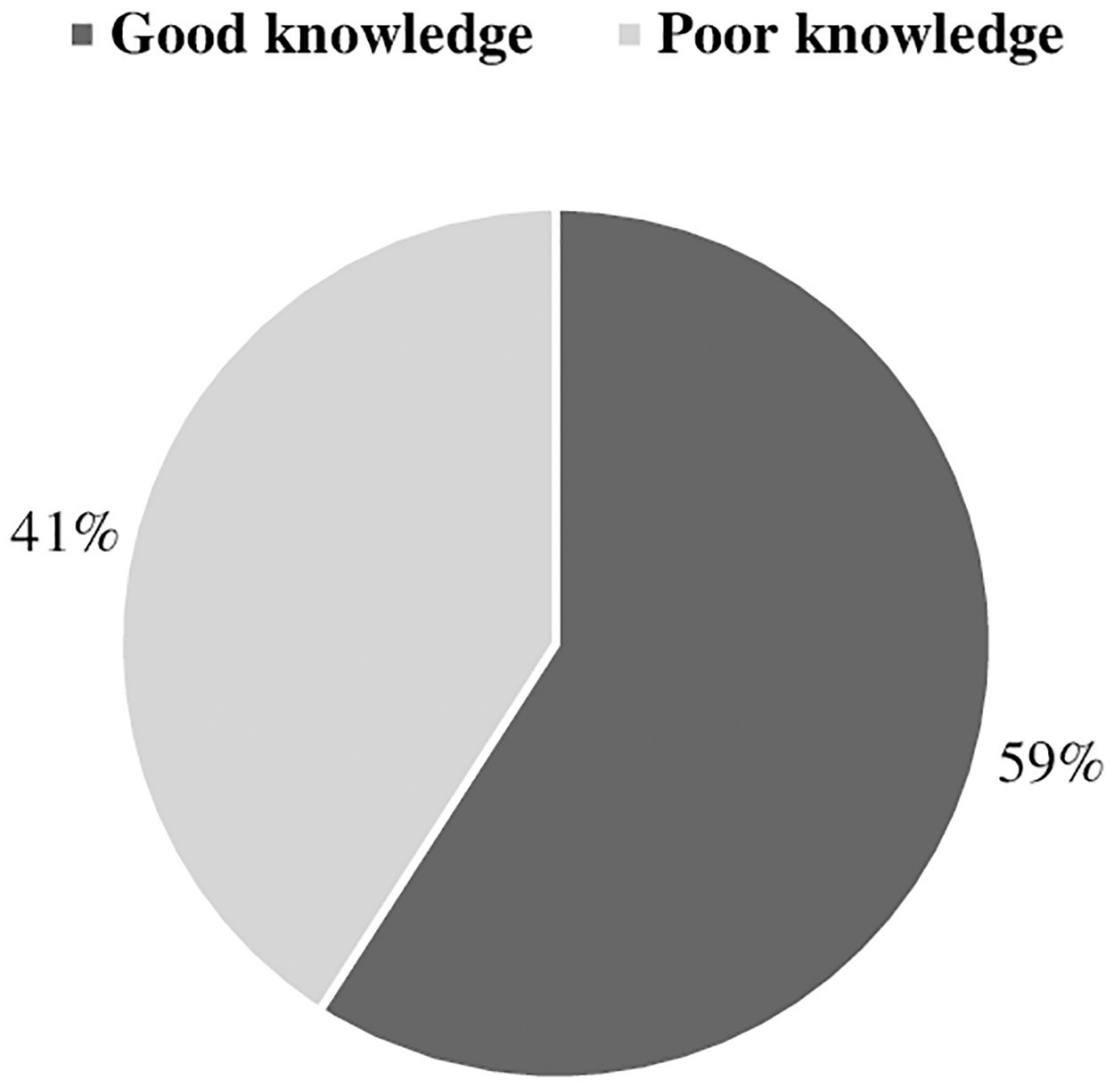
Knowledge about migraine.

In this study, 59.1% (n = 1167) of the participants had a good level of knowledge about migraines, while 40.9% (n = 808) had a poor level of knowledge (Fig 4). The knowledge score for migraine was significantly associated with gender (*p* = 0.002), age (*p* = 0.0001), educational level (*p* = 0.001), employment status (*p* = 0.001), monthly income (*p* = 0.0001), region *(p* = 0.0001), and history of migraine (*p* = 0.0001). However, the migraine knowledge score was not significantly correlated with nationality (*p* = 0.793). A detailed explanation of the correlations between the knowledge scores and the participants’ demographic characteristics is provided in Table 4.

**Table 4:** Association between participants’ knowledge score and their demographic characteristics.

Demographics	Good knowledge	Poor knowledge	*p*-value
	n (%)	n (%)	
**Gender**			0.002
Male	782 (67%)	486 (60.1%)	
Female	385 (33%)	322 (39.9%)	
**Age (years)**			0.0001
18–24	85 (7.3%)	508 (62.9%)	
25–33	582 (49.9%)	155 (19.2%)	
34–51	420 (36.0%)	104 (12.9%)	
52–64	49 (4.2%)	28 (3.5%)	
>64	31 (2.7%)	13 (1.6%)	
**Nationality**			0.793
Saudi	1166 (99.9%)	807 (99.9%)	
Non-Saudi	1 (0.1%)	1 (0.1%)	
**Educational level**			0.0001
Secondary	319 (27.3%)	159 (19.7%)	
University	616 (52.8%)	458 (56.7%)	
Postgraduate	116 (9.9%)	103 (12.7%)	
Intermediate	116 (9.9%)	88 (10.9%)	
**Employment status**			0.0001
Housewife	227 (19.5%)	88 (10.9%)	
Businessman	386 (33.1%)	77 (9.5%)	
Student	70 (6.0%)	477 (59.0%)	
Unemployed	145 (12.4%)	70 (8.7%)	
Retired	66 (5.7%)	31 (3.8%)	
Employed	273 (23.4%)	65 (8.0%)	
**Monthly income (SAR)**			0.0001
Good (10,000–15,000)	548 (47.0%)	181 (22.4%)	
Low (<5K SAR)	135 (11.6%)	188 (23.3%)	
Average (5,000–9,999)	363 (31.1%)	354 (43.8%)	
Excellent (>15,000 SAR)	121 (10.4%)	85 (10.5%)	
**Region**			0.0001
Central region	88 (7.5%)	64 (7.9%)	
Western region	116 (9.9%)	230 (28.5%)	
Northern region	294 (25.2%)	103 (12.7%)	
Eastern region	253 (21.7%)	252 (31.2%)	
Southern region	416 (35.6%)	159 (19.7%)	
**History of migraine**			0.0001
Yes	723 (62.0%)	627 (77.6%)	
No	444 (38.0%)	181 (22.4%)	

To determine the relationship between the knowledge score for migraine and gender, age, nationality, education, employment, income, and region where the participants were living, a multiple regression linear model was utilized in which gender, age, education, nationality, employment, income, and region were considered as explanatory variables and the knowledge score for migraine as the dependent variable. These results revealed that the participants’ gender (95% Cl = -.653 to -.236, p = < .001), age, education, employment, income, and region of residence were significant predictors of the knowledge score (p < .001), as shown in Table 5. However, the participants’ nationality did not affect the knowledge score for migraine, as shown in Table 5.

**Table 5:** Multiple linear regression analysis predicting the knowledge score for migraine.

Variables	Unstandardized coefficients		Standardized coefficients	*t*	*p*-value	95.0% Confidence interval for B	
	B	Std. Error	Beta			Lower bound	Upper bound
(Constant)	.930	1.550		.600	.549	-2.110	3.969
Gender	-.444	.106	-.082	-4.182	< .001	-.653	-.236
Age (years)	1.497	.055	.520	27.014	< .001	1.389	1.606
Nationality	-1.251	1.524	-.015	-.820	.412	-4.240	1.739
Educational level	-.136	.056	-.046	-2.431	.015	-.246	-.026
Employment status	.097	.031	.061	3.091	.002	.036	.159
Monthly income (SAR)	-.234	.047	-.095	-5.001	< .001	-.326	-.142
Region	.166	.038	.082	4.325	< .001	.091	.242

## Discussion

Nationally and internationally, there was a shortage of literature on migraine knowledge and perceptions; students and healthcare professionals had provided the vast majority of the available information. This research will significantly benefit patients and people in Saudi Arabia and other nations in terms of migraine prevention and control, and it will serve as a reference for desperately needed future studies.

In this study, 68.4% of individuals experienced migraines. For example, a prior study in 2021 by Bamalan et al. among the general population residing in Jeddah reported a prevalence of 37.2% [8], while the prevalence in the United States was 15.3% and adults in the United States had an age-adjusted prevalence of 15.9% [8]. A recent systematic analysis of migraine in Arab nations, on the other hand, found that the prevalence ranged from 2.6% to 32% [9]. Another study from a developing nation found a lifetime migraine prevalence of 25.2% [40].

Photophobia was the most often mentioned migraine symptom in this study, followed by headaches, vomiting, nausea, and visual loss. These findings were consistent with previous research by other authors [41, 42]. For example, El-Metwally et al. reported that the most common causes of migraines were anxiety, depression, and hypertension [9], whereas another study found that the most frequently experienced symptoms were extreme sensitivity to light and sound, as well as vomiting or nausea [43].

It was found that 59.1% of the participants had a good understanding of migraines overall. Furthermore, the male participants (67%) knew the most. A study conducted by Aljunaid et al. found that 45.6% of healthcare practitioners were adequately knowledgeable about migraines [44]. Furthermore, previous studies have indicated that increased awareness was associated with improved knowledge and perceptions of specific diseases [33, 35, 45]. Disease knowledge varies from study to study and is influenced by a variety of factors such as the methodology employed and the respondents’ demographic characteristics.

In this study, one-third of the participants thought that genetic disease was the main cause of migraine, followed by physical disease, head trauma, and psychiatric diseases. According to Algahtani et al. and El-Metwally et al. [1, 9], 17.14% of their participants believed that genetic disorders, organic diseases, psychiatric disorders, and head trauma caused migraines [1]. Furthermore, it has been found that migraines are also triggered by sleep disturbances, prolonged exposure to excessive sunlight or heat, fatigue, hunger, and stress [9]. Additionally, another study found that migraine headaches were triggered by individuals’ age, a low level of education, socioeconomic conditions, head injury, obesity, chronic stress, and excessive caffeine and medication consumption [46].

In terms of the age groups affected by migraine, the current findings show that the elderly (67.9%) are the most common age group affected by migraine. Almost half (49.1%) of the participants believed that both genders were equally affected, while 115 of them thought that migraines affected females. An earlier study reported similar findings [1] that migraines affect people of all ages and they do not affect one gender more than the other. Only 86 participants (22.3%) were aware that young adults were affected by migraines, and 76 participants (19.7%) believed that the disease affected females more than males. The history of migraine in women might be due to the differences in hormones or the presence of the female hormone (estrogen), which is less commonly found in men. This might be the reason why women have more episodes of migraine than men. However, a previous report suggested that as women begin to menstruate, and their hormones begin to fluctuate, their migraines dramatically increase in number [47]. Furthermore, there are reports in the literature that life events, stress, and neuronal activity all play a role. All of these are interconnected and they influence one another. This sex phenotype for women should be taken into account during clinical management and experimental studies [47].

Migraine sufferers’ quality of life is greatly affected by migraines, which result in headache-related disability, a lower socioeconomic status, and a lower level of health-related quality of life. This in turn increases the comorbid and psychiatric conditions associated with the disease, and it results in increased use of healthcare resources and higher healthcare-related costs [48]. Furthermore, studies have indicated that the average headache-related total healthcare costs for migraine sufferers are more than three times those of other patients [8]. Migraines also have a significant impact on the social, physical, emotional, and personal lives of those who suffer from them [49, 50]. In addition to causing significant cognitive impairments, which have a negative impact on daily activities such as academic, work-related, and sports performance, they also impose a significant burden on society if they are not treated or properly managed [50].

The findings of this study, revealing a high prevalence of migraines and significant variations in participants’ knowledge, underscore the urgent need for targeted policy interventions in Saudi Arabia. Policy makers should prioritize comprehensive educational programs to enhance awareness among healthcare professionals and the public, emphasizing the causes, symptoms, and treatment options for migraines. Addressing demographic differences, particularly the higher prevalence among the elderly and females, requires personalized healthcare strategies incorporated into national policies. Such measures can contribute to improved pharmaceutical practices, better patient outcomes, and a reduced social burden of migraines. This study provides a foundational resource for evidence-based policies, encouraging a comprehensive approach to migraine management and potentially inspiring similar initiatives globally.

There are some limitations to this study. It is possible that biases such as recollection bias or social desirability bias may have been a factor since the results were based on a self-administered questionnaire. The convenience sampling in this study may introduce bias, as participants were easily accessible, limiting the generalizability of findings to the broader migraine population. The findings were limited to Saudi Arabia, rendering them unrepresentative of other countries. While acknowledging the study’s limitation of its limited focus on Saudi Arabia, future research should consider expanding the geographic scope for cross-cultural comparisons. Combined international efforts could provide a global perspective on migraine patterns. Additionally, investigating pediatric migraine prevalence, adopting a longitudinal approach, and exploring cultural determinants within the Saudi context are suggested to enhance our understanding and inform targeted interventions.

## Conclusions

In this study, one-third of the participants were found to have good knowledge of migraines, yet there was a knowledge deficiency in certain clinical aspects of migraines. It is important to note that the prevalence of migraines is rising and affecting a considerable percentage of the Saudi population. This finding suggests that efforts are needed to reduce and control the incidence of migraine and its adverse events by providing adequate treatment options. Furthermore, this research will act as a reference for the much-needed future research and it can be used to create a health education program to raise public awareness about migraines. It is essential to educate the community and provide them with the most up-to-date and accurate information to improve their knowledge and awareness about the disease and its negative consequences.
